# Pattern Recognition Receptor Signaling and Innate Responses to Influenza A Viruses in the Mallard Duck, Compared to Humans and Chickens

**DOI:** 10.3389/fcimb.2020.00209

**Published:** 2020-05-12

**Authors:** Lee K. Campbell, Katharine E. Magor

**Affiliations:** ^1^Department of Biological Sciences, University of Alberta, Edmonton, AB, Canada; ^2^Li Ka Shing Institute of Virology, University of Alberta, Edmonton, AB, Canada

**Keywords:** influenza, duck, reservoir host, innate immunity, tropism

## Abstract

Mallard ducks are a natural host and reservoir of avian Influenza A viruses. While most influenza strains can replicate in mallards, the virus typically does not cause substantial disease in this host. Mallards are often resistant to disease caused by highly pathogenic avian influenza viruses, while the same strains can cause severe infection in humans, chickens, and even other species of ducks, resulting in systemic spread of the virus and even death. The differences in influenza detection and antiviral effectors responsible for limiting damage in the mallards are largely unknown. Domestic mallards have an early and robust innate response to infection that seems to limit replication and clear highly pathogenic strains. The regulation and timing of the response to influenza also seems to circumvent damage done by a prolonged or dysregulated immune response. Rapid initiation of innate immune responses depends on viral recognition by pattern recognition receptors (PRRs) expressed in tissues where the virus replicates. RIG-like receptors (RLRs), Toll-like receptors (TLRs), and Nod-like receptors (NLRs) are all important influenza sensors in mammals during infection. Ducks utilize many of the same PRRs to detect influenza, namely RIG-I, TLR7, and TLR3 and their downstream adaptors. Ducks also express many of the same signal transduction proteins including TBK1, TRIF, and TRAF3. Some antiviral effectors expressed downstream of these signaling pathways inhibit influenza replication in ducks. In this review, we summarize the recent advances in our understanding of influenza recognition and response through duck PRRs and their adaptors. We compare basal tissue expression and regulation of these signaling components in birds, to better understand what contributes to influenza resistance in the duck.

## Introduction

Influenza A virus (IAV) is a negative sense single stranded RNA (-ssRNA) virus which causes significant disease in both humans and animals. Due to rapid accumulation of mutations during replication, this virus can change surface proteins quickly, thus escape both natural and vaccine-based immunity. These mutations also affect the pathogenicity of individual viral strains. In chickens especially, IAV can cause severe disease and mortality. The virus is classified as low pathogenic or highly pathogenic avian influenza (LPAI and HPAI, respectively) depending on the severity of disease that it causes in chickens (Alexander et al., 1986; Burggraaf et al., 2014). LPAI strains cause mild symptoms and the birds generally recover within a few days whereas HPAI strains tend to spread systemically and often kill chickens within the first few days of infection.

IAV preferentially replicates in different tissues and organs in the host, and initial infection often depends on the linkage type of terminal sialic acid on glycoproteins expressed on the surface of cells. Viral hemagglutinin (HA) surface proteins bind to glycoprotein-linked sialic acid (SA) on the surface of host cells. The specific linkage of these sialic acids allows the virus to not only become specific to different host species, but also different tissues in these hosts. Humans express sialic acid α-2,6 linked galactose (SA α-2,6-Gal) surface molecules on epithelial cells in the upper airways, which is the site of replication for IAV in humans (Baum and Paulson, 1990; Couceiro et al., 1993). As such, strains of IAV that infect humans replicate in the upper airways. Birds, however, predominantly express SA α-2,3-Gal in their digestive tracts and lungs (Costa et al., 2012). Strains of IAV which are adapted to replicate in birds preferentially bind these receptors over human SA α-2,6-Gal receptors. Chickens also express α-2,6-Gal in their intestinal tracts and lungs, whereas ducks only express these receptors in their lungs. Chickens also have a predominance of SA α-2,6-Gal in their trachea whereas ducks have SA α-2,3-Gal receptor dominance (Kuchipudi et al., 2009). As IAV has been known to jump host species, as is the case of avian IAV jumping to humans, this suggests that chickens may be responsible for propagating avian strains of influenza that can then infect humans. IAV can use other receptors such as phosphoglycans on host cells to gain entry and seems to depend on more than just SA linkages to enter cells (Byrd-Leotis et al., 2019).

Ducks and migratory waterfowl are thought to be the reservoir hosts of IAV, as they appear to have shared a long evolutionary history with the virus (Webster et al., 1992; Taubenberger and Kash, 2010). Indeed, phylogenetic analysis has suggested that avian IAV and circulating mammalian strains of IAV share a recent common ancestor of avian origin. So called “dabbling ducks,” or more specifically ducks of the genus *Anas*, are the most frequent host of circulating strains of IAV (Kida et al., 1980; Olsen et al., 2006; Runstadler et al., 2007; Jourdain et al., 2010). For simplicity, we will generalize the term “ducks” to mean mallard ducks (*Anas platyrhynchos*), which also includes the many breeds of domesticated mallard ducks (Zhang et al., 2018). When infected with IAV, ducks generally have no or very mild symptoms, yet surprisingly still replicate and excrete viruses at high titres (Kida et al., 1980). LPAI can replicate in the intestines of ducks for up to 5 days without causing lesions (Daoust et al., 2013). Often called the “Trojan Horse” of infection, these migratory birds can then spread the virus to other ducks in waterways, or to other bird species as they migrate (Kim et al., 2009). HPAI however, preferentially replicates in the lungs of infected ducks, and is more likely to spread systemically in ducks and chickens (Bingham et al., 2009; Vidana et al., 2018). After such a long evolutionary history, the reservoir host likely has evolved adaptations to circumvent damaging effects of prolonged viral replication.

While ducks can control most strains of IAV, some HPAI strains cause significant disease and mortality in ducks, especially those belonging to the H5 subgroup and clade 2.3.2.1 (Sturm-Ramirez et al., 2004; Bingham et al., 2009; Hagag et al., 2015; Haider et al., 2017). It is difficult to generalize, however, because in challenge experiments using viruses belonging to this clade, ducks demonstrated differences in mortality ranging from 100% lethal to no mortality (Kang et al., 2013; Ducatez et al., 2017). Most strikingly, two viruses from the 2.3.2.1 clade that differed by only 30 amino acids showed complete differences in mortality in mallards, with one virus being 100% lethal while the other causing no mortality (Hu et al., 2013). All of these strains are lethal to chickens and many other species. However, some species may show resistance to some strains. Pigeons are resistant to some strains of H5N1, including to strains belonging to clade 2.3.2 (Smietanka et al., 2011; Yamamoto et al., 2012). However, as summarized in a recent review (Abolnik, 2014), pigeons often do not replicate the virus to significant titres and only shed the virus for a short period of time. We also cannot generalize about all ducks as other types of ducks exhibit varied reactions when infected with H5N1 strains of virus. Gadwall, wigeon, and mallard ducks were asymptomatic, while mandarin duck, tufted ducks, ruddy shelducks, and several species of geese and swans showed signs of morbidity and mortality (Gaidet et al., 2010). In another study, swans and ruddy shelducks showed 100% mortality when infected with HPAI H5N1, whereas mallard ducks had an asymptomatic infection (Kwon et al., 2010). Thus, infection and mortality rates differ between different types of ducks. These studies highlight the difficulty in making generalizations about avian influenza studies but can also pinpoint residues contributing to virulence in each host species. What makes mallard ducks so successful at both limiting viral replication of HPAI virus and resisting damage from replicating virus is currently unknown.

When birds are infected with IAV, the first few days seem to be the most important when determining survival vs. succumbing to infection, highlighting the importance of innate immunity as a protective mechanism. We recently reviewed the immune responses of ducks and chickens to IAV (Evseev and Magor, 2019). Birds diverged from mammals about 300 million years ago yet have retained many of the same innate immune mechanisms that mammals use to combat viral infections. When viral or pathogen associated molecular patterns (PAMPS) are detected by the host, they are detected by specific pattern recognition receptors (PRRs) in order to elicit antiviral responses including cytokines, chemokines, and upregulation of antiviral effectors. Both immune and non-immune cells contain these PRRs. PRRs of avian species were previously reviewed in 2013 (Chen et al., 2013), however significant advances have been made since that time. In this review, we summarize recent advances in understanding innate signaling pathways in ducks by looking at the similarities and differences between PRR tissue expression in ducks, chickens, and humans. We also further review new research in characterizing protein function in the signal transduction platform in order to understand how innate signaling pathways differ or are the same in these three species.

**Figure 1:**
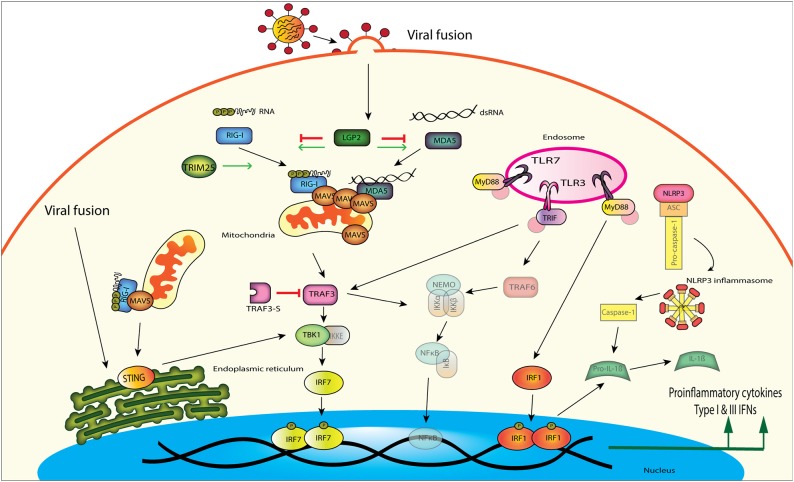
Influenza A virus is detected by several different innate immune signaling proteins in the cell. RIG-I and MDA5 both can bind to viral RNA and signal downstream through MAVS, TBK1, and IRF7. Viral fusion can also be detected by the endoplasmic reticulum localized STING, which then signals through TBK1 to induce interferon production. TLRs located in the endosome can recognize viral RNA and signal through different adaptor proteins to induce proinflammatory cytokines and IFNs. TLR3 uses TRIF as an adaptor protein to amplify signaling through TRAF3 or TRAF6, while TLR7 uses the adaptor MyD88 to signal through TRAF3. TLR signaling through MyD88 can also activate the NLRP3 inflammasome, which increases pro-inflammatory cytokine production through IRF1.

The three important PRR signaling pathways responding to influenza infection include toll-like receptors (TLRs), retinoic acid-inducible gene-I (RIG-I)-like receptors (RLRs), and nucleotide-binding oligomerization domain (NOD)-like receptors (NLRs) (Figure 1). TLRs, RLRs, and NLRs can all be found on the cell surface or in cytosolic compartments in the cell. These PRRs all act to recognize influenza viral components such as double stranded RNA (dsRNA), single stranded RNA (ssRNA), and RNA with a 5′ triphosphate overhang (5′pppRNA) (Yoneyama et al., 2004; Okamoto et al., 2017). Many of these PRRs have signaling pathways that converge downstream to produce interferons (IFNs) or proinflammatory cytokines and utilize similar scaffolding and adaptor proteins to amplify this signal. In this review, we compile recent studies on characterization of these influenza sensors, signaling pathways and their downstream effectors in both chickens and ducks.

**Figure 2:**
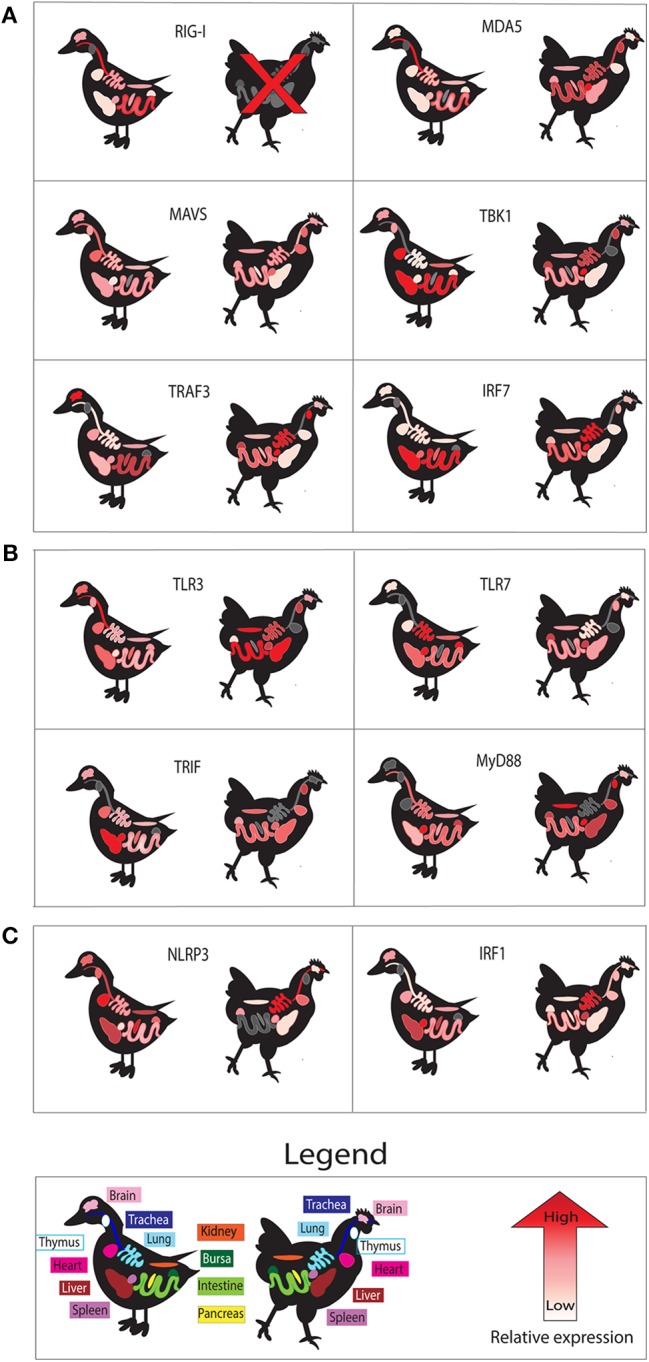
Basal tissue expression of genes in uninfected ducks compared to those in chickens. Tissue expression is shown for components of RLR **(A)**, TLR **(B)**, and NLRP3 inflammasome **(C)** signaling pathways. We show relative expression of each gene studied in those tissues. High relative basal gene expression is denoted by red, while lower expression is indicated by pinks and whites. Gray coloring indicates no data available for the gene in the indicated tissue. All data was extracted from individual studies in this review, and color scales are relative for data from individual studies. All data is for mallard duck, except RIG-I and MDA5, which are Muscovy duck. Data obtained for chicken MDA5, MAVS, and IRF1 were obtained from the chicken atlas (http://biogps.org/), and averages for each tissue in adult chickens were used to estimate relative expression.

Basal expression of these PRRs may also allow different tissues to detect IAV infection earlier. To visualize PRR readiness we show basal expression patterns in different tissues in ducks and chickens (Figure 2). Tissues studied include immune relevant organs such as the lung, spleen, bursa, thymus, and intestine as well as other organs such as brain, kidney, and heart.

## RLR Receptors and Their Adaptors

The RIG-I like receptor (RLR) family are select cytosolic RNA helicases which contain conserved DExD/H box domains used in nucleic acid binding (Loo and Gale, 2011). These PRRs sense non-self RNA from viral pathogens. In contrast to other PRRs like TLRS, RLRs are expressed in immune cells as well as in somatic cell types such as epithelium, thus can protect cell types most targeted by viral infection (Uhlen et al., 2015; Francisco et al., 2019). RLRs involved in IAV recognition include retinoic acid inducible gene I (RIG-I), melanoma differentiation-associated gene 5 (MDA5), and laboratory of genetics and physiology 2 (LGP2) (Figure 1). RIG-I and MDA5 share much structural similarity, with both proteins having two caspase activation and recruitment (CARD) domains, a central DEAD helicase domain and a C-terminal repressor domain (RD) (Yoneyama et al., 2005; Zou et al., 2009). While the DExD/H box helicase domain has the ability to use ATP hydrolysis to aid in binding and unwinding viral RNA, the RD has been implicated in self repression (as in RIG-I). CARD domains are involved in relaying the signal to the downstream adaptor, the mitochondrial antiviral signaling protein (MAVS) (Jacobs and Coyne, 2013; Wu and Hur, 2015). LPG2 is lacking the CARD domains that RIG-I and MDA5 possess but shares structural similarity in the DExD/H box and RD domains (Pippig et al., 2009). The cytosolic sensor MDA5 preferentially recognizes long dsRNA, whereas RIG-I recognizes shorter dsRNA sequences that are produced during IAV replication (Kato et al., 2008). Once these cytosolic sensors recognize viral RNA, a signal is transduced through MAVS to downstream components to induce type I IFN or proinflammatory cytokine production.

### RIG-I

RIG-I is the primary sensor of influenza virus in all cells except plasmacytoid dendritic cells. RIG-I detects dsRNA and viral transcriptional intermediates bearing 5′-pppRNA in infected cells (Hornung et al., 2006; Pichlmair et al., 2006; Schmidt et al., 2009). A panhandle structure, formed by binding of complementary regions in the influenza RNA transcript, is detected by RIG-I (Liu G. et al., 2015). Recently transcriptional intermediates called mini viral RNAs of about 80 nucleotides in length have been shown to act as RIG-I ligands (te Velthuis et al., 2018). Notably, it was recently shown that RIG-I detects viral replication not only in the cytoplasm, but also in a nuclear compartment (Liu G. et al., 2018). This may be particularly relevant for influenza detection, since influenza replicates in the nucleus. A recent review considers how dsRNA and viral transcriptional intermediates bearing 5′-pppRNA made in the nucleus are detected by RIG-I in the cytoplasm of infected cells (Liu and Zhou, 2019). It is not known whether RIG-I is capable of nuclear detection in lower vertebrates. In addition, RIG-I (but not MDA5) can act as an antiviral effector protein by directly binding to incoming IAV viral RNA (Weber et al., 2015). RIG-I also has far reaching effects on immune responses. Mice deficient in RIG-I signaling show defects in dendritic cell activation and mobilization, viral antigen presentation and impairment of polyfunctional T cell responses (Kandasamy et al., 2016). More recently, the importance of RIG-I in IAV infection has been questioned. Surprisingly, when RIG-I was knocked out of mice, this did not make mice more susceptible to lethal influenza infection (Wu et al., 2018). These results may stem from mice not being a natural host of IAV or perhaps they rely on different recognition strategies to detect virus.

RIG-I is ubiquitously expressed in human tissues, but ducks have tissue specific basal expression of RIG-I and chickens appear to be missing RIG-I entirely. RIG-I is expressed in most human tissues and does not exhibit tissue specific expression, although there is slightly higher mRNA expression in the thymus, granulocytes, and adipose tissues (Uhlen et al., 2015). A comparison of tissue expression of RLR pathway components between chickens and ducks illustrates the readiness of these tissues to respond to pathogens (Figure 2A). In Muscovy ducks, RIG-I is most highly expressed in the trachea and digestive tissues (Cheng et al., 2015a). Chickens appear to have lost RIG-I (Barber et al., 2010). RIG-I gene loss has also been documented in mammals, such as the Chinese tree shrew (Xu et al., 2016). RIG-I knockouts generated in C57BL/6 mice are lethal in the developing embryos (Kato et al., 2005), however this lethality was not seen in mice with a more complex genetic background (Wu et al., 2018).

Duck RIG-I can function in chicken cells, indicating that chickens have the corresponding downstream signaling components. When we overexpressed duck RIG-I in chicken fibroblasts, the cells could detect RIG-I ligand and produce interferon (Barber et al., 2010). We also showed that chicken cells transfected with duck RIG-I produce more IFN-β, augment expression of numerous ISGs, and restrict influenza virus (Barber et al., 2013). Others have demonstrated that chickens detect IAV through the related RLR, MDA5 (Karpala et al., 2011; Liniger et al., 2012). We have speculated that one reason ducks so successfully control influenza virus while chickens do not is partially because of RIG-I. This has been controversial, and we acknowledge that because RIG-I has not been detected does not prove it does not exist. No disrupted gene has been found to confirm its absence. If chicken RIG-I has significantly diverged from duck RIG-I, it would not be detected through hybridization, or PCR. Likewise, it has also been notably absent from the now extensive transcriptome databases available for chickens and other galliform birds. However, if a chicken RIG-I ortholog is expressed in very low amounts or has a very high GC content, it may be difficult to sequence using standard next generation sequencing technology. An interesting experiment to determine the significance of RIG-I in birds would be to knock RIG-I out of ducks or introduce duck RIG-I into chickens. However, some strains of influenza viruses can kill ducks even in the presence of RIG-I, demonstrating that many other factors contribute to successful defense.

RIG-I is upregulated quickly during influenza infection, with a peak at 24 h and expression returning to normal levels in lung, intestine, and spleen when Pekin ducks are infected with both HPAI and LPAI IAV strains (Fleming-Canepa et al., 2019). In these studies, RIG-I is upregulated much more during HPAI infection than LPAI infection. In Muscovy ducks RIG-I mRNA expression peaked at 2 DPI in brain and spleen, while expression was highest 1 DPI in the lung and bursa (Cheng et al., 2015a). Muscovy ducks are more susceptible to influenza infection than mallard ducks (Phuong do et al., 2011), and this slight delay in RIG-I upregulation may contribute.

### MDA5

MDA5 was often thought to be of less importance in IAV infection because of its preference for longer dsRNA, however siRNA knockdown of this host mRNA during IAV infection in mice demonstrated that MDA5 is also an important factor in viral restriction (Benitez et al., 2015). While it appears that chickens have lost RIG-I (Barber et al., 2010), they use the related cytosolic receptor MDA5 to detect IAV and signal through MAVS to induce IFN and proinflammatory cytokine responses (Karpala et al., 2011; Liniger et al., 2012). The tree shrew lineage also appears to have lost RIG-I, and pathogen pressures on tree shrew MDA5 and LGP2 have selected for the ability to detect the RIG-I agonist Sendai virus (SeV) (Xu et al., 2016). Chicken MDA5, unlike mammalian MDA5, preferentially recognizes short dsRNA (Hayashi et al., 2014), and like human MDA5 it can also be stimulated with long polyinosinic-polycytidylic acid (poly (I:C) (Barber et al., 2010). It is currently unknown if duck MDA5 has a dsRNA length preference. Chicken MDA5 also appears to have undergone positive selection, and is able to recognize RNA from Newcastle Disease virus (NDV) (Xu et al., 2019). Indeed, when these mutations were introduced into human MDA5, a glutamic acid to a leucine at position 633, the mutant was able to bind NDV RNA. Duck MDA5 has proline at residue 633 (Barber et al., 2010), and thus is not expected to detect NDV RNA.

MDA5 is most highly expressed in the trachea followed by the ileum, duodenum, crop, rectum, and colon in Muscovy ducks (Wei et al., 2014), like basal expression of RIG-I (Cheng et al., 2015a). In healthy adult chickens, MDA5 was most highly expressed in the spleen, followed by the thymus and trachea (Bush et al., 2018). Chicken MDA5 is strongly upregulated in lung, spleen and brain in H5N1 infected birds (Karpala et al., 2011). Duck MDA5 is upregulated in response to IAV infection at 1 DPI in the lung, spleen, and brain, and returns to normal levels at 3 DPI (Wei et al., 2014; Fleming-Canepa et al., 2019). MDA5 was also slightly upregulated in lungs of Pekin ducks infected with LPAI, but not significantly upregulated in intestines of the same cohort of ducks (Fleming-Canepa et al., 2019).

### LGP2

LGP2 is induced in humans during influenza infection. LGP2 seems to function as both a positive and negative regulator of RIG-I and MDA5. This contrary effect on IFN signaling seems to be dose dependent as smaller amounts of LGP2 help increase MDA5 and RIG-I activation while over-expression of LGP2 inhibits it (Rothenfusser et al., 2005; Satoh et al., 2010). In mice infected with IAV, LGP2 attenuates the IFN response, perhaps in an effort to control damaging inflammatory responses (Malur et al., 2012). Recently LGP2 has also been implicated in inhibition of Dicer dependent processing of dsRNA, thus inhibiting RNAi (van der Veen et al., 2018). Muscovy ducks infected with HPAI H5N1 had upregulation of duck LPG2 (duLGP2) in the spleen at 1 DPI (Jiao et al., 2015). In the lung and brain, duLGP2 was upregulated on both 1 and 2 DPI suggesting that duLGP2 is involved in the early response to IAV. This is the same expression pattern seen in geese infected with this strain of H5N1 (Wei L. et al., 2016). No studies have been published to date on duLGP2 interactions with RIG-I during IAV infection. However, duLGP2 was important during duck enteritis virus (DEV) infection through interactions with MDA5 (Huo et al., 2019). Overexpression of chicken LGP2 (chLGP2) reduced IFN signaling in IAV infected cells, however silencing of the LGP2 gene in chicken cells also decreased IFN-β production, suggesting chLGP2 is important for MDA5 signal enhancement at low expression levels (Liniger et al., 2012). It is unknown if duLGP2 augments signaling with duck RIG-I or MDA5.

### TRIM25

Tripartite motif protein 25 (TRIM25) can both augment IFN signaling (Gack et al., 2007) and directly restrict virus in mammals (Meyerson et al., 2017). TRIM25 is known to stabilize RIG-I CARD domain interaction with MAVS CARD domains and increase IFN production during an infection (Gack et al., 2007). The CARD domains of RIG-I are exposed when RIG-I recognizes viral RNA, at which point TRIM25 binds to RIG-I CARD domains using its C-terminal PRY-SPRY domain. Using the E3 ligase activity of its RING domain, TRIM25 polyubiquitinates RIG-I, attaching K63-linked ubiquitin chains to lysine residues on RIG-I. Stabilization of the RIG-I CARD domain tetramer allows it to nucleate MAVS filament formation (Peisley et al., 2014). TRIM25 can also physically block vRNA transcription in the nucleus by binding to the vRNP complex (Meyerson et al., 2017). Whether duck TRIM25 has the ability to restrict viral RNA transcription not yet been examined.

Duck TRIM25 performs much the same function as human TRIM25 in RIG-I stabilization. Human TRIM25 ubiquitinates lysine 172 of human RIG-I CARD domains, but this lysine is not conserved in ducks. Instead, duck TRIM25 ubiquitinates K167 and K193 (Miranzo-Navarro and Magor, 2014). Mutation of either lysine site alone in the duck did not alter ubiquitination patterns of the CARD domains, however mutation of both sites abrogated covalently attached ubiquitin. Interestingly, duck TRIM25 in our transfection experiments could still activate these double mutants, suggesting unanchored ubiquitin could also stabilize RIG-I in the duck. Chicken TRIM25 augments IFN signaling, however the mechanism is unclear in the absence of RIG-I (Rajsbaum et al., 2012). In human cells, a long non-coding RNA (lncRNA) Lnczc3h7a also contributes to stabilizing the interaction between TRIM25 and RIG-I CARD domains (Lin et al., 2019). Recently, duck lncRNA were analyzed during HPAI and LPAI infection to determine which were differentially expressed and potentially involved in influenza A control (Lu et al., 2019). This study did not assess whether lnczc3h7a is differentially expressed, nor is it known if duck lnczc3h7a can function in the same manner, but this augmentation by lncRNAs may well be conserved.

In healthy chickens, TRIM25 is most highly expressed in the lung, spleen, and thymus and is upregulated in response to NDV in the spleen, thymus, and bursa (Feng et al., 2015). To date, we are unaware of studies looking at TRIM25 basal tissue expression in duck, however we showed TRIM25 is upregulated in the lung of HPAI infected ducks and slightly upregulated in lung of LPAI infected ducks at 1 DPI (Fleming-Canepa et al., 2019).

### MAVS

MAVS protein is an adaptor protein that acts as a signaling amplifier during viral infection through interactions with both RIG-I and MDA5 (Figure 1). MAVS forms “prion-like” aggregates on the surface of the mitochondria when nucleated by tetramers of CARD domains of RIG-I or MDA5 (Kawai et al., 2005; Hou et al., 2011). The 2CARD domains of RIG-I form a helical tetrameric structure offset by 1 unit, and this helical assembly recruits MAVS CARD monomers (Wu et al., 2014). The helical assembly of tetrameric RIG-I and elongation of MAVS filaments is necessary for signal transduction by MAVS. Although ducks have very different amino acid sequences within these CARD domains compared to mammals, we showed the helical assembly of d2CARD with MAVS leads to signal activation as well (Wu et al., 2014). Filamentous MAVs then recruits tumor necrosis factor receptor associated factor 3 (TRAF3), which acts as an adaptor protein to phosphorylate TANK-binding kinase 1 (TBK1) and inhibitor of nuclear factor-κB (IκB) kinase (IKK) (Fitzgerald et al., 2003; Liu S. et al., 2015). From there transcription factors such as interferon regulatory factor 3 or 7 (IRF3/IRF7) are activated to induce IFN production.

Duck MAVS expression in healthy tissues varied depending on the age of the ducks tested. In 3-week old Cherry Valley ducks, MAVS expression was highest in the pancreas, liver and heart (Li N. et al., 2016), while in 2-month-old Cherry Valley ducks, tissue expression was more ubiquitous with slightly higher expression seen in the trachea and heart (Li H. et al., 2016). MAVS basal expression in adult chickens is also more ubiquitous, with only slighter higher expression seen in the spleen, heart, and thymus (Bush et al., 2018). The human protein atlas shows that human MAVS is expressed in almost all tissues, but curiously has the lowest expression in innate immune cells such as dendritic cells, monocytes, T-cells and B-cells (Uhlen et al., 2015). Pekin duck MAVS is upregulated 1 DPI in both HPAI and LPAI infection in lungs, however no MAVS upregulation was seen in ileum of LPAI infected ducks (Fleming-Canepa et al., 2019).

### TBK1

TBK1 activates IFN-β production by phosphorylating IRF3 allowing it to dimerize and translocate to the nucleus and initiate type I IFN production (Fitzgerald et al., 2003; Liu S. et al., 2015) (Figure 1). In humans TBK1 (huTBK1) expression is highest in brain tissues, adrenal glands, lungs, and the upper digestive tract (Uhlen et al., 2015). Chickens express TBK1 highest in spleen, lung, and thymus (Wang et al., 2017). This contrasts with 1-month old Cherry Valley ducks, where the highest expression was seen in the liver, heart, and duodenum (Hua et al., 2018). Very little expression was seen in healthy lungs, spleen, or bursa of these ducks. Duck TBK1 (duTBK1) was shown to function similarly to huTBK1 in that overexpression was able to activate IFN-β, NF-κB, and IRF1 promoter activity in duck embryonic fibroblast (DEF) cells. Silencing of endogenous duTBK1 in DEF cells also significantly reduced IFN-β promoter activity in DEF cells. As basal tissue expression of many duck PRR and downstream signaling components seems to favor having reduced expression of these proteins in immune relevant sites, we suggest that this could be another level of immune regulation that is protective to the duck. Experimental dysregulation of basal tissue expression of proteins such as TBK1 and IRF7 could be done to investigate this question.

### TRAF3

TRAF3 operates downstream of both TLRs as well as RLRs to aid in signal transduction and amplification (Hacker et al., 2006) (Figure 1). In the RIG-I signaling pathway, TRAF3 acts as an adaptor downstream of MAVS, by recruiting TBK1 and IKKε to phosphorylate the transcription factor IRF3 (Guo and Cheng, 2007). TRAF3 is most highly expressed in lung, spleen, and thymus of 2-week-old chickens (Yang et al., 2015). Duck TRAF3 (duTRAF3) however, has a uniform expression pattern with only slightly higher amounts of TRAF3 expression seen in the brain, and the lowest levels in the lung (Wei et al., 2018). In chicken embryonic fibroblasts (CEF) cells, TRAF3 (chTRAF3) is upregulated in response to poly (I:C) stimulation, NDV infection and poly dA-dT, suggesting it is important in both DNA and RNA viral infections (Yang et al., 2015). Similarly, duTRAF3 is also upregulated in DEF cells stimulated with poly (I:C) and the authors also found that overexpression of duTRAF3 could control both IAV and duck Tembusu virus replication (Wei et al., 2018).

Curiously, a truncated version of duTRAF3 was also found, named duTRAF3-S (splice isoform duck TRAF3) (Wei et al., 2018). This splice variant is missing key N-terminal catalytic domains but can still bind to both TBK1 and MAVS with its C-terminal TRAF domain. DuTRAF3-S can interact with duTRAF3 but not MAVS, thus decreasing IFN-β production. After poly (I:C) stimulation, DEF cells express more duTRAF3 until 9 HPI, at which point duTRAF3 mRNA expression begins to decrease and duTRAF3-S mRNA expression begins to increase. This splice isoform may act to dampen IFN signaling in the later time points of infection to reduce damage from inflammation. In summary, duTRAF3 is most highly expressed in the brain in healthy ducks, while chickens express more in the lung, spleen, and thymus.

### IRF7

IRF3 is a known important mediator of the type I interferon system in mammals. IRF3 is ubiquitously expressed, slow to degrade and a potent transcriptional activator of Type I IFN production in mammals (Honda and Taniguchi, 2006). Birds appear to be missing IRF3, however, they do have IRF7 (Cormican et al., 2009; Huang et al., 2010). Avian IRF7 is structurally like IRF3, suggesting that it may play a similar role to that of IRF3 in mammals. Recent bioinformatics analysis has confirmed that chicken IRF7 clusters more closely to IRF3 of lower vertebrates, yet is located in a region with high synteny to mammalian IRF7 (Cheng et al., 2019b).

IRF3, rather than IRF7, is considered more important for the initial response to viral infection. In mammals IRF3 is constitutively expressed in most tissues and seems to have a long half-life (Prakash and Levy, 2006; Hiscott, 2007). Activation of IRF3 results in increased type I IFN signaling and an eventual increase in transcription of IRF7, which has a very short half-life, comparatively. IRF7, in turn, amplifies both Type I and Type III IFN signaling (Sato et al., 1998). In mice, knockdown of IRF3 is not detrimental to the IFN response to IAV, however knockdown of IRF7 leaves mice much more susceptible to infection and a double knockout of both transcription factors renders mice unable to produce IFN-α or IFN-β (Hatesuer et al., 2017). Humans who have mutations in IRF7 are more susceptible to life threatening infections by IAV (Ciancanelli et al., 2015). There is very little expression of duck IRF7 (duIRF7) in the lung of uninfected ducks, and greater expression seen in the liver and intestine (Chen et al., 2019). Chicken IRF7 (chIRF7) is most highly expressed in the spleen and lung of healthy chickens (Cheng et al., 2019b).

Recent research has focused on the role of IRF7 in inhibition of IAV through IFN mediated responses in chickens and ducks. Chicken IRF7 (chIRF7) is involved in antiviral responses and plays analogous roles to that of mammalian IRF3. Recent studies have found that chIRF7 can be induced to translocate across the nucleus downstream of both chMAVS and chicken stimulator of interferon genes (chSTING), and chIRF7 dimerizes following chTBK1 activation, allowing it to increase IFN-β signaling (Wang et al., 2019). Initial experiments investigating function found that overexpression of chIRF7 increased IFN-β expression (Kim and Zhou, 2015). However, their knockdown of chIRF7 did not significantly change IFN-β expression during poly (I:C) stimulation suggesting other transcription factors may be involved. Contradictory results were published in 2019 showing chirf7^−/−^ DF-1 cells were unable to produce IFN-β, even when transfected with MAVS or STING (Cheng et al., 2019b). DuIRF7 upregulates type I IFNs but does not affect type II IFN expression (Chen et al., 2019). We showed that duIRF7 increases IFN-β signaling when overexpressed in DF-1 cells (Xiao et al., 2018). We also observed duIRF7 translocate to the nucleus upon stimulation with constitutively active RIG-I 2CARD. When chIRF7 is overexpressed in DF-1 cells, it caused increased cell death and resulted in higher levels of viral replication (Kim and Zhou, 2018). With transfection of mCherry-IRF7 (Xiao et al., 2018), we also observed increased cell death.

IRF7 can control viral replication in ducks. A recent study demonstrated that duIRF7 can control the positive sense RNA virus, duck Tembusu virus in DEF cells (Chen et al., 2019). No studies to date have examined whether duIRF7 controls IAV, or if it increases viral replication, as seen in DF-1 cells. This may be an interesting avenue of study, as Kim and Zhou (2018) suggest that chIRF7 could be a target of IAV.

### STING

Stimulator of interferon gene (STING) is a protein on which many PRR pathways converge in order to increase NF-κB and IFN signaling downstream of pathogen pattern recognition (Figure 1). It was initially discovered as an adaptor molecule in the cyclic GMP-AMP synthase (cGAS) signaling pathway, which detects viral DNA and subsequently drives the induction of type I IFNs and proinflammatory cytokines (Ishikawa et al., 2009). STING also interacts with both RIG-I and MAVS in mammalian cells and is involved with sensing of RNA viruses (Zhong et al., 2008; Castanier et al., 2010). STING is found on the endoplasmic reticulum and can be closely associated to MAVS on the mitochondrial outer membrane (Zhong et al., 2008; Ishikawa et al., 2009). Acting as a scaffolding protein between TBK1 and IRF3, STING aids in IRF3 phosphorylation and type I IFN induction (Zhong et al., 2008; Tanaka and Chen, 2012). IAV interferes with STING through its hemagglutinin fusion peptide, effectively preventing STING dimerization and interactions with TBK1 (Holm et al., 2016). In addition, independently of RIG-I or TLR detection, STING also detects RNA viral membrane fusion events and potentiates the IFN response during viral infection (Holm et al., 2012).

Duck STING (DuSTING) shares 43 and 71% identity to human and chicken STING (chSTING), respectively (Cheng et al., 2019a). DuSTING is most highly expressed in the glandular stomach, followed by the trachea, lung, small intestine, spleen, kidney, and bursa (Cheng et al., 2019a). ChSTING is most highly expressed in the thymus, bursa, spleen, lung, and intestine of uninfected chickens (Ran et al., 2018). As chSTING was not analyzed in the glandular stomach or trachea, it is not possible compare expression to ducks. However, it is noteworthy that in ducks, STING is more abundant in the lung than the bursa and spleen. If duSTING is orthologous to mammalian STING, it may react to IAV fusion quicker in these tissues although it is not known if duSTING can detect viral fusion. Human STING shows low tissue specific expression, but has slightly higher mRNA expression in tonsils, lymph nodes, and lung (Uhlen et al., 2015).

Human STING increases IFN-β signaling when overexpressed in 293 T cells (Ishikawa et al., 2009). MEF cells were shown to require STING but not cGAS to produce IFN after infection with two RNA viruses, NDV and SeV. Similarly, duSTING drastically increased IFN-β promoter activation when overexpressed in DEF cells. However, when the cells were stimulated with poly (I:C), STING was not required to potentiate the IFN response (Holm et al., 2016). DuSTING is highly upregulated in both spleen and lung in ducks infected with a LPAI H9N2. DuSTING was most highly upregulated on day 2 in both these tissues. In lungs, duSTING was only upregulated on day 2, with day 1 showing no significant increase when compared to mock infected birds (Cheng et al., 2019a). This may be because a LPAI strain of virus was used. It would be interesting to look at STING regulation in these tissues during HPAI infection.

## Toll-Like Receptor Pathway

TLRs are important pattern recognition receptors that induce innate immune responses to viral, bacterial, fungal and parasitic pathogens (Kawai and Akira, 2010). Humans have 10 TLRs (TLR1-10) as do birds, however the TLRs that have been classified in birds are different, as reviewed by several groups (Boyd et al., 2007; Temperley et al., 2008; Brownlie and Allan, 2011; Chen et al., 2013; Keestra et al., 2013). For example, TLR1 in birds has been duplicated so that birds express TLR1a and TLR1b. Similarly, TLR2 has two paralogous genes, *tlr2a* and *tlr2b*. Other homologous TLRs expressed by birds include TLR3, TLR4, TLR5, and TLR7, which leaves TLR8, TLR9, and TLR10 currently unaccounted for in avian species. Birds also have two TLRs which are not found in mammals but have been classified in lower vertebrates: TLR15 and TLR21. TLR15 is upregulated in response to bacterial pathogens in chickens (Nerren et al., 2010), and recognizes a yeast-derived agonist (Boyd et al., 2012) and diacylated lipopeptide from mycoplasma (Oven et al., 2013). TLR21 functions analogously to TLR9 in humans in that it recognizes CpG oligodeoxynucleotides (CpG ODN) in both duck (Cheng D. et al., 2019) and chicken (Brownlie et al., 2009).

TLRs can be expressed both extra and intracellularly, with the cell surface TLRs being more adept at detecting extracellular pathogens (TLR1, 2, 4, 5, and 6) (Hopkins and Sriskandan, 2005). Likewise, TLRs that are in endosomes, or in other intracellular compartments, are more specialized in detecting intracellular pathogens, such as viruses (TLR3, 7, 8, and 9). Specific TLRs, such as TLR3, TLR7, and TLR8 recognize viral RNA and play important roles in the defense against IAV in mammals (Alexopoulou et al., 2001).

PAMPs are detected through the TLR ectodomain with leucine rich repeats (LRR) and signal downstream to produce IFNs and other cytokines through their cytoplasmic Toll/IL-1 receptor (TIR) domain (Botos et al., 2011). TLRs are activated and different adaptor proteins are recruited to amplify the signal. TIR-domain-containing adapter-inducing interferon-β (TRIF) dependent pathways induce type I IFN production through TBK1 and IRF3 activation (Sato et al., 2003; Yamamoto et al., 2003). Myeloid differentiation primary response 88 (MyD88) dependent pathways induce NF-κB proinflammatory gene expression through recruitment of TRAF6 and eventual activation of the IKK signaling complex (Hemmi et al., 2002; Muroi and Tanamoto, 2008).

Induction of TLR signaling increases IFN production and cytokine signaling in both mammalian and avian cells. As such, treatment of cells with TLR specific ligands such as poly (I:C), lipopolysaccharide (LPS) and CpG ODN can reduce IAV replication in both mammals (Cluff et al., 2005; Shinya et al., 2011) and chickens (St. Paul et al., 2012; Barjesteh et al., 2014). TLRs can also act synergistically to produce proinflammatory responses. In chicken monocytes, stimulating with the TLR3 ligand poly (I:C) resulted in an increase in mRNA of type I IFNS (He et al., 2012). Co-stimulation of these chicken monocytes with the TLR21 ligand CpG-ODN and poly (I:C) resulted in an even greater increase of proinflammatory cytokines than cells stimulated with a single ligand and biased the cells to a Th1 type response. Since only TLR3 and 7 directly detect IAV during infection in birds, we will focus on these TLRs in the next two sections.

### TLR3

TLR3 is an endosomal TLR that recognizes dsRNA or replicating viral intermediates and activates NF-κB signaling in a TRIF dependent signaling pathway (Alexopoulou et al., 2001) (Figure 1). In humans, TLR3 is predominantly expressed in the placenta, followed by smaller but still significant amounts in the small intestine and lower amounts in most other tissues (Uhlen et al., 2015). It is also constitutively expressed in bronchial and alveolar epithelial cells (Guillot et al., 2005). Infection of the human cell line A549 (alveolar epithelial cell line) with IAV resulted in an upregulation of TLR3 (Wu et al., 2015). TLR3 stimulation during influenza infection resulted in activation of IRF3 and increased type III IFN production. When TLR3 knockout mice were infected with influenza they had a surprising survival advantage over wildtype mice, despite having higher viral titres in their lungs (Le Goffic et al., 2006), highlighting the complex role of this PRR in influenza restriction.

Tissue expression of TLR3 differs between ducks and chickens (Figure 2B). In uninfected tissues, Pekin duck TLR3 is expressed highest in the trachea with lower expression seen in the digestive tissues and the lung (Zhang M. et al., 2015). Muscovy ducks, which are more susceptible to influenza virus infection than Pekin or mallard ducks (Pantin-Jackwood et al., 2013) show higher expression of TLR3 in the trachea, spleen, pancreas, lung, and digestive tissues (Jiao et al., 2012). Thus, Muscovy ducks show high basal expression of TLR3 in many tissues, while Pekin ducks had high expression only in trachea. In chickens, basal TLR3 expression is highest in intestine, liver, and kidney (Iqbal et al., 2005). TLR3 was constitutively expressed in chicken heterophils (Kogut et al., 2005).

After infection with HPAI virus, Muscovy duck TLR3 was upregulated at 24 HPI in the lung and brain, with sustained expression in the brain (even though this is a non-fatal infection in Muscovy ducks) (Jiao et al., 2012). There was no increased expression in the spleen. In contrast, transcriptomic data from Shaoxin mallard ducks infected with a HPAI H5N1 show increased TLR3 expression in the lungs, peaking on day 2 of infection (Huang et al., 2013). This discrepancy between the Muscovy duck and Shaoxin mallard TLR3 expression data may be due to the strains of virus used in the infection (DK212 vs. DK49; both H5N1) but not age of the birds as both experiments used 4-week old ducks. Chickens upregulated TLR3 in the lung during HPAI H5N1 infection when replicating virus was still present in lung tissues (Ranaware et al., 2016). In reovirus infected ducks, TLR3 expression peaked at 72 HPI in the lung, while spleen and bursa showed a sustained response from 24 to 48 h (Zhang M. et al., 2015). These results are of interest as Reovirus infection in Muscovy duck can cause mortality in 20–40% of infected animals (Malkinson et al., 1981; Wozniakowski et al., 2014).

### TRIF

TRIF is the adaptor molecule downstream of TLR3 and TLR4 and provides a signaling platform to recruit other adaptor proteins and increase type I IFNs and proinflammatory cytokine expression (Figure 1). Similar to humans (Yamamoto et al., 2003), in uninfected tissues, ducks express TRIF most highly in the pancreas and spleen (Wei X. et al., 2016) (Figure 2B). Chicken TRIF expression was found to be highest in the cecum, heart, liver, spleen, and kidney (Wheaton et al., 2007). Expression of duck TRIF peaks at 12 h after treatment with poly (I:C), however, it peaks much later at 36 h post infection with IAV (Wei X. et al., 2016), likely due to viral suppression of IFN signaling pathways in infected cells.

### TLR7

Human TLR7 produces a robust type I IFN response upon detection of IAV or other ssRNA viruses using the MyD88-dependent pathway (Diebold et al., 2004; Lund et al., 2004). TLR7 is highly expressed by murine plasmacytoid dendritic cells (pDCs) and is located in endosomal compartments where it can detect incoming viral RNA (Diebold et al., 2004), and produce high levels of IFN-α. RNA from live and inactivated influenza virus can be detected by TLR7 in the endosome of pDCs, provided the hemagglutinin remains intact for receptor-mediated viral entry (Diebold et al., 2004) TLR7 detection is thus known to induce IFN-α, and proinflammatory cytokines (Figure 1). Suggesting that the role of TLR7 and RIG-I signaling is complicated in influenza infection, *Tlr7*^−/−^*Mavs*^−/−^ knockout mice succumb quickly to a lethal influenza infection as expected, however infection with a low viral dose revealed that proinflammatory signaling promoted viral replication by recruiting susceptible monocytes (Pang et al., 2013). Oddly, humans have enhanced tissue expression of TLR7 in the brain, with lower expression in mucosal tissues (Uhlen et al., 2015).

Tissue expression of TLR7 is notably different between healthy ducks and chickens (Figure 2B). Duck TLR7 is expressed the highest in spleen, bursa, and lung (MacDonald et al., 2008; Kannaki et al., 2018). In chickens, basal TLR7 expression is highest in spleen, bursa, and intestine with very little expression in the lung (Iqbal et al., 2005; Philbin et al., 2005), initially suggesting that this distribution may play a role in chicken susceptibility to HPAI strains that replicate in the lungs. However, the chicken macrophage cell line HD11 expresses high levels of TLR7 (Philbin et al., 2005), and both primary macrophages and heterophils constitutively express TLR7 in other studies (Kogut et al., 2005). The chicken atlas on the BioGPS server agrees with the previous studies in that TLR7 expression is limited in the lung, and higher in tissues such as the spleen, bursa, and immune cells (Bush et al., 2018). It is however worth noting that TLR7 basal expression in chickens is slightly variable depending on the breed and age of chicken sampled. Stimulation using TLR7 agonists decreased viral replication in chicken macrophages (Stewart et al., 2012; Barjesteh et al., 2014; Abdul-Cader et al., 2018), indicating TLR7 can induce IFNs in those cell types. Thus, chicken strains may vary with respect to TLR7 expression. Ducks infected with HPAI upregulate TLR7 most highly in their lungs 2 DPI while chickens infected with the same virus had only a slight increase in expression at 1 DPI (Cornelissen et al., 2013). In contrast, ducks infected with a LPAI H7N9 had only marginal upregulation of TLR7 In their lungs 0.8 DPI, while chickens had a significant increase in this expression 0.8 DPI (Cornelissen et al., 2012).

### MyD88

MyD88 conveys the signal downstream of most of the TLRs, to induce an inflammatory response upon detection of pathogens (Figure 1). MyD88 signaling was found to be important for protecting mice during primary influenza infection, as MyD88^−/−^ knockout mice were more susceptible (Seo et al., 2010). MyD88 may also be an important factor in initiating damaging cytokine storms in the host, since there was a significant reduction in proinflammatory cytokines and activated macrophages and neutrophils in the lungs of MyD88^−/−^ mice, but not TRIF^−/−^ mice, following IAV infection (Teijaro et al., 2014). Ducks have two isoforms of the *myd88* gene that have been characterized, named DuMyD88-X1 and DuMyD88-X2 (Cheng et al., 2015b). DuMyD88-X2 is a truncated version that encodes a premature stop codon and produces a protein with an interruption in the TIR signaling domain. DuMyD88-X1 is highly expressed in uninfected ducks in all immune relevant tissues including the lung, intestine, and bursa, but it showed the strongest expression in the spleen (Figure 2B). DuMyD88-X2 was expressed in these same tissues but to a much lower extent than the X1 isoform. Both isoforms of MyD88 could activate the IL-6 promoter and induce NF-κB activity in duck cells. In ducks challenged with NDV, the X1 isoform was upregulated in liver and spleen. Neither isoform was as highly expressed in the lung during NDV infection, and no studies have looked at the expression of these genes during influenza infection. Three isoforms of MyD88 have been found in chickens (named MyD88-1, 2, and 3) (Qiu et al., 2008). Chicken MyD88 (chMyD88) is the largest of the isoforms, and is ubiquitously expressed, which agrees with previous research on chMyD88 expression although it is of note that these studies demonstrated slightly more chMyD88 expression in the thymus, liver, and spleen than in other tissues tested (Wheaton et al., 2007). ChMyD88 is not significantly upregulated in DF-1 cells infected with influenza (Barber et al., 2013). Upregulation in influenza-infected chicken tissues has not been explored, but MyD88 is upregulated by LPS treatment (Wheaton et al., 2007). As MyD88 plays a role in immune system derived damage during influenza infection in mammals, it would be interesting to know if chMyD88 activation is significantly different from the duck.

## NLR Receptors—the NLRP3 Inflammasome

### NLRP3

The NOD-like receptor family pyrin domain containing 3 (NLRP3) can form multi protein complex inflammasomes, which possess autocatalytic activity. This activity can activate caspase-1 and induce the production of proinflammatory cytokines IL-1β and IL-18 (Figure 1). NLRP3 inflammasome induction can occur in immune cells such as macrophages (Pirhonen et al., 2001) and dendritic cells (Fernandez et al., 2016) and as well in other cell types such as fibroblasts and epithelial cells (Allen et al., 2009; Pothlichet et al., 2013). Deletion of NLRP3 in mice causes a decrease in immune cell recruitment to the site of infection and poor outcomes when infected with influenza (Allen et al., 2009; Thomas et al., 2009).

Tissue expression of NLRP3 differs between ducks and chickens (Figure 2C). NLRP3 is fairly ubiquitously expressed in healthy chicken tissues but most highly expressed in chicken trachea and lung (Ye et al., 2015). Duck NLRP3, however, is most highly expressed in the pancreas with very low expression in the lung and slightly higher expression in the trachea (Li et al., 2018). This expression profile is of interest as NLRP3 inflammasome activation has been associated with contributing to cytokine storms and severe pathology from influenza infection (Teijaro et al., 2014). We are unaware of studies detailing the NLRP3 inflammasome response to influenza infection in either chicken or duck.

### IRF1

IRF1 is known to be an activator of IFNs though several mechanisms, but one of importance is its regulation of the NLRP3 inflammasome (Kuriakose et al., 2018) (Figure 1). It is thought that by regulating the NLRP3 inflammasome, IRF1 contributes to apoptosis and necroptosis during influenza infection. Kuchipudi et al. (2012) suggest that duck cells are more likely to become apoptotic when infected with IAV than chicken cells. Indeed, DEF cells infected with HPAI strains that are known to cause severe symptoms in infected ducks had decreased apoptosis (Kuchipudi et al., 2012). Thus, IRF1 as a regulator of early apoptotic response is an interesting candidate to study in ducks. Human IRF1 is expressed highest in the spleen and the liver (Uhlen et al., 2015). Duck IRF1 (duIRF1) is most highly expressed in liver and spleen, followed by the pancreas, and digestive tissues such as the stomach and duodenum. Interestingly, it is expressed in very low levels in the lung and trachea (Qian et al., 2018) (Figure 2C). The chicken atlas on the BioGPS server indicates that chicken IRF1 (chIRF1) expression in healthy adult birds is highest in the lung, spleen, and thymus (Bush et al., 2018).

Overexpression of chIRF1 in DF-1 cells caused a significant increase of IFN-β, Mx, and MDA5 mRNA (Liu Y. et al., 2018). chIRF1 mRNA also substantially increased 12 HPI after infection with either IAV or NDV. These transcripts rapidly dropped back down to basal levels after 12 h. Poly (I:C) stimulation of duck fibroblasts resulted in duIRF1 transcripts peaking at 12 HPI and then decreasing, as in chicken cells. However, when these cells were infected with H5N1 the duIRF1 mRNA began to increase at 12 HPI and continued to increase until 48 HPI. The delay in the duck response may be due to strain differences between viruses used (Qian et al., 2018) as the chIRF1 study used A/Chicken/Shanghai/010/2008 (H9N2) while the duIRF1 study used A/Duck/Hubei/hangmei01/2006 (H5N1). DuIRF1 interacts with MyD88 to increase IFN-β independently of IRF7, and overexpression of duIRF1 not only upregulated Type I IFNs but also Type III IFN (IFN-λ) (Qian et al., 2018). When ducks were infected with H6N2, duIRF1 transcripts peaked at 36 HPI, rather late in infection compared to other ISGs or IFNs mentioned in this article. As duIRF1 does not signal downstream of RIG-I, it could be used as a secondary pathway to limit viral replication. Overexpression of duIRF1 also limited H9N6 and H5N1 viral replication.

## Interferon Responses and ISGS

### Type I IFNs

Type I interferons include IFN-α and IFN-β, both which are present in birds (Santhakumar et al., 2017). Airway epithelium, macrophages, and pDC are responsible for most of the type I IFNs produced during viral infection (Onoguchi et al., 2007; Khaitov et al., 2009; Crotta et al., 2013). Plasmacytoid dendritic cells are known to produce much of the initial IFN-α (Ito et al., 2005; Liu, 2005), and it is thought that the autocrine action of IFN-α on the pDCs upregulates antiviral factors such as Mx1 and thus protects against influenza infection (Cella et al., 1999). An early IFN response generally provides more positive outcomes in infection, and studies have also implicated type I IFN responses as a factor that can reduce pro-inflammatory cytokine release and thus limit damage (Billiau, 2006; Guarda et al., 2011; Arimori et al., 2013).

Both transcriptomic and qPCR studies have demonstrated that ducks have a robust but short response of type I IFNs in response to HPAI (Cagle et al., 2011; Vanderven et al., 2012; Saito et al., 2018). Transcriptomic data demonstrated that lungs of ducks infected with a HPAI H5N1 strain had an increase in *IFNA* expression days 1 and 2 DPI (Huang et al., 2013). While IFNs are most strongly upregulated within the first 24 h, it should be noted that many ISGs have a sustained response for up to 3 DPI (Huang et al., 2013; Smith et al., 2015). Ducks infected with HPAI H5N1 strains A/goose/Guangdong/16568/2016 (GS16568), and A/duck/Guangdong/16873/2016 (DK16873) showed sustained responses of type I IFNs post infection. However, the time points used in these experiments were 12 HPI and 2 DPI (Wu et al., 2019). While these highly pathogenic strains of flu could be eliciting sustained responses, other strains of H5N1 had the peak of IFN upregulation at 1 DPI (Saito et al., 2018). LPAI induces a relatively weak IFN response in ileum of infected ducks (Vanderven et al., 2012).

In ducks infected with HPAI H5N1 strains VN1203 and D4AT, we found that IFN-α and IFN-β were most upregulated 1 DPI in lungs and spleens of infected birds (Saito et al., 2018). The spleen had a greater increase in IFN-α transcripts compared to the lung, while lung showed higher upregulation of IFN-β. This may reflect the relative contribution of different PRRs in these tissues; while TLRs are largely responsible for IFN-α, IFN-β expression is largely RIG-I dependent (Opitz et al., 2007). By day 2 the IFN response had been reduced to mock infection levels. When testing the expression of IFN-α in primary avian cells infected with either H5N1 or H5N9, it was highest in duck cells at 12 and 24 HPI (Jiang et al., 2011). In chicken and turkey cells, IFN-α was most highly expressed at 24 HPI.

Pre-treatment with IFN-α protects duck cells, but not adult ducks from IAV infection. DEF cells treated with IFN-α show a reduced viral load as well as induction of many ISGs (Gao et al., 2018b). Interestingly, pre-treatment of primary chicken lung cells and duck fibroblasts with IFN-α before infection with IAV reduced IFN-α production in both these cell types (Jiang et al., 2011). The protective effects of IFN-α seem to be age dependent in the duck. When looking at survival rates of 2 days vs. 3 weeks old ducklings treated with rIFN-α before infection of HPAI H5N1, the treatment with IFN benefited the 2 days old ducklings but not the 3 weeks old ducks (Gao et al., 2018b). The rIFN-α dose may have been insufficient to protect the older ducks, or alternatively IFN-α is not protective. In contrast, 7 and 33-day old chickens treated with rIFN-α before exposure to a chicken-isolate H9N3 were both found to be protected (Meng et al., 2011). These results are of interest, as generally younger ducks are more susceptible to IAV infection, and protection correlates with onset of RIG-I expression (Londt et al., 2010; Pantin-Jackwood et al., 2012). The DK383 H5N1 virus used, which is lethal in ducks (Gao et al., 2018b), may impair RIG-I signaling, and IFN-α alone is not sufficient to protect the older ducks. Similarly, IFNB knockout mice are much more sensitive to influenza, suggesting IFN-α cannot fully compensate (Koerner et al., 2007). These results seem to support the hypothesis that an early and quick response is more beneficial to the duck than a sustained type I IFN response.

### Type II Interferons

IFN-γ is classified as a type II IFN and is secreted by NK cells, CD8^+^ lymphocytes and CD4^+^ T helper cells (Schroder et al., 2004). While IFN-γ has been found in some studies to be protective against influenza (Weiss et al., 2010), other researchers have shown that by knocking out the genes or knocking down gene expression in mice, absence of IFN-γ protected the mice from severe infection with pandemic H1N1 (Califano et al., 2018). Similarly, other studies in mice have shown that IFN-γ negatively regulates the survival of CD8+ T cells during influenza infection and limits the number of influenza specific memory cells available during an infection (Prabhu et al., 2013).

CEFs treated with IFN-γ were more resistant to infection by H9N2 avian influenza virus and H1N1 human influenza virus. Stimulation with IFN-γ also increased IFN-α/β, and Mx transcripts in these cells (Yuk et al., 2016). Likewise, DEF cells treated with recombinant duck IFN-γ showed significant decreases in viral replication with a HPAI H5N1. Two-day old ducks were pre-treated with IFN-γ before being infected with DK383 IAV serotype H5N1. In these experiments 6/10 ducks that were pre-treated survived the infection at 10 DPI, while in PBS treated controls only 2/10 ducks survived (Gao et al., 2018a). As age played a factor in IFN-α pre-treatment reducing viral load in ducks, it would be worthwhile to repeat these experiments in older ducks. To our knowledge no studies have investigated whether duck IFN-γ influences the development of memory T cells during IAV infection.

### Type III Interferons

Type III IFNs induce an antiviral state like that of type I IFNs but use different receptors for detection. Additionally, type III IFN receptors are expressed predominantly in airway epithelial cells and intestinal epithelia (Sommereyns et al., 2008), unlike type I IFN receptors, which are more ubiquitously expressed. Ducks and chickens express one kind of type III IFN (IFN-λ) (Karpala et al., 2008; Yao et al., 2014; Santhakumar et al., 2017) whereas other vertebrates produce one to four different type III IFNs, depending on the species (Kotenko et al., 2003; Chen et al., 2016).

Primary CEF and DEF cells both produce IFN-λ (chIFN-λ and duIFN-λ, respectively) in response to both poly (I:C) stimulation and infection with a mouse-adapted strain of H1N1 (Zhang Z. et al., 2015). Interestingly, DEF cells produce less IFN-λ transcripts when stimulated with poly (I:C) or infected with H1N1 than CEF cells. These same DEF cells also highly upregulate IFN-λ receptor transcripts at 36 HPI whereas the CEF cells highly express the receptor transcripts at 8 HPI and continue to do so until 36 HPI. A separate study found that chIFN-λ was unable to induce an antiviral state in the chicken fibroblast DF-1 cell line when infected with a HPAI H5N1, indeed the cells were not able to respond to recombinant chIFN-λ until they were transfected with the receptor (Reuter et al., 2014). This discrepancy may be due to the use of primary cells in one study and an immortalized cell line in the other. Immortalized cells often drastically change genotype and so the DF-1 cells may have stopped expressing the chIFN-λ receptor. High levels of the chIFN-λ receptor transcripts were found in the lung, trachea and intestine (Zhang Z. et al., 2015), suggesting that like chIFN-λ receptor expression is like that of humans. There is currently very little research on duIFN-λ, its receptor or antiviral activity, making this a promising candidate for future studies into IAV resistance in the duck.

## Other Antiviral Proteins of Interest

### TRIM Proteins

TRIM proteins are a large family of intracellular proteins with diverse functions such as cell cycle regulation, autophagy, proteasomal degradation, development, and immunity which have been comprehensively reviewed (van Gent et al., 2018). Most interestingly, some of these proteins allow species-specific protection from viruses through viral restriction. One of the first TRIM proteins discovered, the alpha isoform of TRIM5 (TRIM5α) was found to restrict HIV in non-human primates, while the human ortholog was unsuccessful in restricting this virus (Stremlau et al., 2004; Sawyer et al., 2005). This highlights the evolutionary relationship these proteins have with pathogens and suggests that members of this protein family might be providing their host species a significant advantage.

A study from 2008 listed 38 TRIM genes in chicken, compared to human, rat, mouse, dog, and cow on their TRIMgene online database (Sardiello et al., 2008). Very few studies have been done on avian TRIM proteins. Avian TRIM25 has a specific role in the activation of RIG-I as discussed above in section TRIM25 (Rajsbaum et al., 2012; Miranzo-Navarro and Magor, 2014). A family of related TRIM genes was discovered in the avian MHC-B locus in both chicken (Ruby et al., 2005; Shiina et al., 2007) and duck (Blaine et al., 2015), with the MHC location suggesting this gene expansion may have arisen from pathogen pressures. The set of TRIM proteins in the MHC-B locus of birds all contain the B30.2/PRYSPRY C-terminal domain motif. Proteins containing this domain have recently expanded in TRIM protein evolution (Sardiello et al., 2008). The PRYSPRY domain is thought to be able to recognize specific amino acid sequences rather than peptide motifs, giving it pathogen specific activity (James et al., 2007; D'Cruz et al., 2013). Ducks also have an expanded butyrophilin gene family, proteins which also contain a B30.2/PRYSPRY domain (Huang et al., 2013).

Of the expanded TRIM genes in the duck MHC, TRIM27.1, and TRIM27-L were found to have antagonistic functions in the MAVS signaling pathway (Blaine et al., 2015). TRIM27-L significantly increased IFN-β signaling in a dose dependent manner while TRIM27.1 slightly decreased this same signaling in DF-1 cells. When co-expressed TRIM27-L activity overrode the inhibition of TRIM27.1. Curiously TRIM27-L appears to have been lost in Galliformes while being retained in Anseriformes, other birds and reptiles. As the Galliformes have also lost RIG-I it seems that either TRIM27-L expression was detrimental and thus lost in evolution or provided no benefit. Further, TRIM27.1 expression is higher in infected tissues than TRIM27-L. As the decrease in IFN-β was only slight, it could be that TRIM27.1 is playing another role in infection. TRIM27.1 may be upregulated to inhibit influenza without influencing cytokine signaling, as TRIM32 does in some human cell types (Fu et al., 2015). Of the chicken MHC-B TRIM genes, only TRIM39 has been cloned and tissue expression analyzed, but no function has been determined (Pan et al., 2011).

TRIM23 was identified as a differentially expressed gene in a microarray study from ducks infected with both HPAI and LPAI strains of IAV, as upregulated 5 DPI in LPAI but not HPAI infections (Kumar et al., 2017). TRIM23 is an ancient TRIM with well-conserved structural homology, and uses its ADP-ribosylation factor (ARF) domain to activate TBK1 through GTPase activity (Sparrer et al., 2017). TBK1 then activates selective autophagy, controlling viral replication. This is an interesting observation as LPAI virus can replicate in ducks for many days past initial infection, and the upregulation of TRIM23 suggests it is worth investigating whether it affects viral replication.

Finally, TRIM62 was identified as a retroviral restricting protein in chicken cells (Li et al., 2019), and until recently TRIM62 was only known to function in innate immune signaling augmentation in fish (Yang et al., 2016). It is not known to be antiviral in mammals. TRIM62 can restrict retroviruses in chickens, but no investigation of anti-IAV potential of this protein has been done in chickens or ducks.

### avIFIT

Interferon-induced proteins with tetratricopeptide repeats (IFITs) are a family of proteins which have diverse functions in the cell such as mediating apoptosis, sequestering viral proteins and cell cycle regulation and have been extensively reviewed (Diamond and Farzan, 2013; Fensterl and Sen, 2015). IFITs have undergone duplication in mammals, fish and frogs, while ducks and chickens only have a single IFIT gene (avIFIT) (Zhou et al., 2013). Evolutionary analysis of duck avIFIT found that it most closely resembled mammalian IFIT5 (Wang et al., 2015; Rong et al., 2018a). Human IFIT5 is effective in restricting RNA virus replication by both interacting with immune signaling components (i.e., RIG-I and MAVS) (Zhang et al., 2013) and by binding 5′-ppp viral RNA (Abbas et al., 2013). In chickens, avIFIT (called IFIT5 by the authors) inhibits viral replication by interacting with 5′-triphosphate viral RNA and blocking subsequent replication steps (Santhakumar et al., 2018), similar to the mechanism of IFIT1 and IFIT5 in mammals (Abbas et al., 2013; Habjan et al., 2013).

Duck avIFIT is constitutively expressed in all tissues at basal levels but shows highest expression in digestive tissues such as intestine and stomach, although the expression levels in these tissues is still relatively low (Wang et al., 2015). To date we are unaware of any data on basal expression levels of avIFIT in the chicken. IFIT5 has low tissue specific expression in humans (Uhlen et al., 2015). Despite the slight differences in expression between humans and ducks, IFIT5/avIFIT is highly upregulated in both these species when induced by IFNs. Similarly, studies have demonstrated that avIFIT is upregulated during influenza infection in chicken intestinal epithelial cells when infected with LPAI (Kaiser et al., 2016) as well as in lungs of chickens infected with HPAI H5N1 (Ranaware et al., 2016).

When both human and chicken IFIT5 were overexpressed in chicken cells, they were found to inhibit viral replication and likewise, when chicken IFIT5 was knocked out from these cells, they were much more susceptible to infection (Santhakumar et al., 2018). Chicken avIFIT is found near the mitochondria in chicken cells, and as human IFIT5 interacts with both RIG-I and MAVS in infected cells, it would be worthwhile to investigate subcellular location of duck avIFIT. DF-1 cells were depleted of chicken avIFIT and transfected with duck avIFIT (Rong et al., 2018a). Duck avIFIT can inhibit IAV in DF-1 cells and was shown to do so by both upregulating IFNα/β and by binding the viral nucleoprotein (NP) from an H5N1 flu strain. This antiviral activity was not limited to only influenza virus, as in these experiments duck avIFIT also restricted double-stranded RNA and DNA viruses. Interestingly, in these DF-1 cells duck avIFIT also arrested cell growth in both infected and uninfected cells.

### Mx

Mx1 is an ISG which is highly upregulated in response to viral infection, whose function and regulation as been recently reviewed (Haller et al., 2015). It acts as an antiviral effector and belongs to a large family of GTPases. Both humans and mice have two Mx genes while birds have one. Mx was found to be protective in laboratory mice, as many lab strains were found to have isoforms of Mx1 with exon deletions that left these mice more susceptible to influenza infection than mice with intact Mx1 (Lindenmann, 1962; Horisberger et al., 1983; Staeheli et al., 1988).

Mx is upregulated strongly in brain, lung and spleen of ducks that show a strong IFN response to infection (Smith et al., 2015; Saito et al., 2018). Mx alleles are highly variable in ducks (Dillon and Runstadler, 2010), however only a few of them have been experimentally analyzed for antiviral function. When transfected into mouse or chicken cells, duck Mx was not able to restrict IAV replication (Bazzigher et al., 1993). Chicken Mx weakly inhibits influenza, and that ability is dependent on the breed of chicken that the Mx was cloned from (Ko et al., 2002; Fulton et al., 2014), indicating high diversity in avian Mx. Chicken Mx also appears to be missing the GTPase activity of mammalian Mx proteins, suggesting this may be why antiviral activity has been weak at best in previous studies (Schusser et al., 2011). While more research on allelic variants and their potential to restrict IAV should be done, it is also possible that due to the close evolutionary relationship between IAV and ducks, the virus has evolved the ability to evade avian Mx during infection.

### OASL

Interferon-inducible 2′-5′-oligoadenylate synthase (OAS) and OAS-like protein (OASL) are two related ISGs in humans, which are known to restrict influenza. OAS senses and degrades dsRNA through synthesis of oligoadenylates, which in turn switches on RNase L activity (Sarkar et al., 1999a,b; Justesen et al., 2000; Silverman and Weiss, 2014). RNase-L then degrades all mRNA in the cell (including ribosomal RNA), thus blocking viral replication. OASL inhibits viral replication independently of enzymatic activity by stabilizing the interaction of RIG-I and MAVS in a similar manner to that of ubiquitinylation by TRIM25. OASL has C-terminal ubiquitin-like domains that stabilize RIG-I CARD oligomers, thus potentiating downstream IFN signaling (Zhu et al., 2014; Ibsen et al., 2015). Birds do not appear to have OAS, but have OASL (Sokawa et al., 1984; Tag-El-Din-Hassan et al., 2018). Unlike human OASL, duck OASL has oligoadenylate synthetase activity, as well as the ability to restrict viral RNA in an RNase L independent manner (Rong et al., 2018b). It appears duck OASL functions as both human OAS and OASL, as it can activate both RNase L and RIG-I pathways. Chicken OASL has been found to inhibit WNV in mammalian cells (Tag-El-Din-Hassan et al., 2012). Chicken OASL is highly upregulated in tracheal epithelial cells 24 HPI (Jang et al., 2015). Both ostrich and duck OASL transfected into chicken DF-1 cells could control replication of both HPAI and LPAI influenza virus (Rong et al., 2018b). When OASL was knocked out of DF-1 cells, the cells became more permissive to influenza infection. Consistent with a role in augmenting innate signaling, overexpression of either ostrich or duck OASL also significantly increased the expression of RNase L, as well as other important immune effectors such as IFNα, IFNβ, IRF1, IRF7, Mx, and PKR.

### PKR

The double-stranded RNA (dsRNA)-dependent protein kinase (PKR) is an ISG which functions as both an antiviral effector and anti-proliferative protein during infection (Garcia et al., 2006). PKR binds foreign dsRNA in the cytoplasm and autophosphorylates in order to become active, at which point it then phosphorylates eukaryotic initiation factor 2 (eIF-2α) causing broad inhibition of protein translation in the cell (Galabru and Hovanessian, 1987; Hovanessian, 1989). PKR has two N-terminal dsRNA-binding domains, which are both able to recognize viral RNA (Nanduri et al., 1998), and one C-terminal kinase domain.

PKR is an important antiviral effector in mice infected with IAV, as shown by the increased fatality rate of PKR knockout mice when infected with the H1N1 strain WSN (Balachandran et al., 2000). Chicken PKR has been functionally characterized and determined to be antiviral against VSV (Ko et al., 2004). Studies have shown that PKR is upregulated significantly during HPAIV H5N1 infection, even in lethal infections in the chicken where IFN production is limited (Daviet et al., 2009). The non-structural protein 1 (NS1) of IAV inhibits IFN responses in cells through interactions with OAS and PKR (Ma et al., 2010). Indeed, NS1 from HPAI H5N1 in a HPAI H7N9 background bound and inhibited PKR in chicken embryos.

PKR is upregulated in ducks infected with both HPAI and (to a lesser extent) LPAI virus (Fleming-Canepa et al., 2019) but to date we are unaware of any studies functionally characterizing duck PKR during influenza infection. We previously thought that ducks appeared to be missing the second dsRNA-binding domain (Fleming-Canepa et al., 2019), also confirmed by another group (Liu W. J. et al., 2018). However, through transcriptomic assembly done in our lab we have since found a transcript of the full-length PKR, which contains the second dsRNA-binding domain previously thought to be missing. This find suggests that ducks may predominantly express a splice variant of PKR missing the dsRNA-binding domain, or that this splice variant is preferentially amplified during PCR. Interestingly, it has been suggested that NS1 needs to bind both the kinase domain of PKR and residues 170–230 to keep PKR in an inactive conformation and prevent it from responding to dsRNA (Li et al., 2006). These residues correspond to the second RNA binding domain and the linker region of the protein. The two variants of duck PKR may allow ducks to respond to viral RNA despite NS1 antagonism. Duck PKR needs to be functionally characterized to determine not only its antiviral potential, but also expression levels of the full-length transcript.

### Viperin

Viperin (RSAD2) is highly induced by Type I IFN, and many RNA virus infections. Viperin inhibits IAV by perturbing lipid rafts and thus inhibiting viral budding (Wang et al., 2007). Duck viperin is most highly expressed in blood, intestine, lung, and spleen in healthy birds (Zhong et al., 2015). Chicken viperin was upregulated in both spleen and lung of IAV infected birds after 24 h (Goossens et al., 2015). It was also upregulated in chicken splenocytes as early as 6 h after poly (I:C) stimulation. In Newcastle disease (NDV) infected ducks, viperin was found to be highly upregulated after 24 h in the blood and peaked in expression in the lung and brain at 72 HPI (Zhong et al., 2015). Viperin is one of the most highly upregulated genes in duck lungs in response to H5N1 HPAI infection (Fleming-Canepa et al., 2019), however, the levels of viperin expression in chickens infected with the same strain of H5N1 was not mentioned (Smith et al., 2015). Ducks also significantly upregulated viperin in response to LPAI in the lung, but curiously not in the ileum (Fleming-Canepa et al., 2019).

### IFITMs

Interferon-inducible transmembrane proteins (IFITMs) are upregulated upon viral infection, and have antiviral activity (Diamond and Farzan, 2013). This viral restriction usually happens during entry in either the early or late endosomes. Human IFITM1, IFITM2, and IFITM3 have all been shown to restrict IAV *in vitro* (Brass et al., 2009). The naming of the avian IFITMs has been complicated by the evolutionary history of gene duplication in this region during speciation, but sites for post-translational modifications identify IFITM3 as the gene next to B4GALNT4 (Smith et al., 2013), and the duck orthologs follow the same synteny (Blyth et al., 2016).

IFITM3 restricts IAV in both duck and chicken cells. Ducks upregulated all IFITMs including IFITM1, IFITM2, and IFITM3 in both lung and ileum during infection with HPAI, whereas chickens showed minimal upregulation of IFITMs (Smith et al., 2015). When duck IFITM1, IFITM2, IFITM3, and IFITM5 were overexpressed in DF-1 cells and challenged with LPAI, only IFITM3 significantly decreased viral infection (Blyth et al., 2016). Chicken IFITM3 is also able to restrict both IAV and lyssa virus in DF-1 cells (Smith et al., 2013). As IFITM1 and IFITM2 also control IAV in humans, it may be that host-pathogen co-evolution has allowed the virus to evade these proteins in ducks. Notably, duck IFITM1 has an insertion in exon 1, which changes the sub-cellular localization of the protein (Blyth et al., 2016), or it would restrict influenza. A 2017 study found that when duck IFITM2 was transfected into DF-1 cells it could control the replication of avian Tembusu virus (Chen et al., 2017). Avian Tembusu virus is a positive sense RNA virus belonging to the Flaviviridae family (Zhang et al., 2017). As IFITM2 restricts this virus but not IAV, it is possible that either the mammalian IFITM2 developed the ability to restrict IAV later in evolution, or that the avian strains we tested have evolved to escape from IFITM2. The upregulation of IFITM2 during IAV infection is most likely due to interferon stimulation and is not virus specific.

## A Note On Missing Genes and Dark DNA

Throughout this review we have spoken about genes that are presumed missing from ducks, chickens or birds in general. Because bird genomes contain many GC rich areas (Hron et al., 2015), they are notoriously hard to amplify using PCR based methods. As such, genes may be presumed missing in next generation sequencing applications, as well as with exploratory PCR based methods. This leaves many genes thought to not exist in birds, simply undiscovered. Such was the case with tumor necrosis factor alpha (TNFα), which for years was thought to not exist in chickens. It was recently cloned and characterized from chickens and found to have very low homology to mammalian orthologs, as well as have a high GC content (Rohde et al., 2018). We have also updated the full-length sequence of PKR that was formerly thought to be missing specific domains. One method to help with finding undiscovered genes from next gen sequencing data is using more advanced *de novo* assembly methods on combined RNAseq data. With advances in NGS technology and new software development to analyze fragmented GC rich RNAseq data, we will be able to better mine transcriptomes for genes as well as gain insight into avian immune system evolution. Furthermore, a wealth of genome information from many avian species is becoming available.

## Conclusions

In this review, we summarize recent advances in understanding PRR in ducks, comparing them to chicken PRR, and analyzing their downstream signaling adaptors. We also investigated tissue expression of these innate immune components to try to gain insight into where these proteins were most expressed. Higher tissue expression of PRRs and their effectors may allow ducks to respond more quickly to IAV in a tissue-specific manner. A rapid and robust response that is quickly dampened could allow ducks to limit damage from inflammatory sequelae. Duck RIG-I and MDA5 are most highly expressed in the trachea, lung and intestines, areas of both HPAI and LPAI influenza replication (Figure 2A). However, the downstream adaptor molecules TBK1, TRAF3 and IRF7 are mostly expressed in digestive tissues with very little basal expression in the lung. This contrasts with chickens, which have high expression of these proteins in the lung. This pattern may circumvent out-of-control inflammatory reactions to HPAI and be protective to the duck, but further investigation is needed to confirm this. Duck TLR3 is most highly expressed in the trachea, while duck TLR7 is highest in the lung, fitting a similar pattern to the RLRs (Figure 2B). There appears to be a similar pattern of low lung expression of the adaptor of TLR3, TRIF, but there is no data on duck tracheal expression of TRIF to confirm this. Likewise, chicken TRIF basal expression has not yet been looked at in respiratory tissues. NLRP3 has high relative expression in chicken lungs, whereas ducks have higher basal expression in their hearts (Figure 2C). It is interesting to note that ducks seem to express PRRs at a high basal level in areas where influenza replicates, but the adaptor molecules are much less expressed in lung and respiratory tissues, the areas of HPAI replication. As more tissues are investigated, transcriptome mining for expression levels of these PRRs and adaptors, where missing, may help a complete picture to emerge.

Throughout this review we summarize the function and regulation of PRRs in chickens, ducks, and humans during IAV infection. While the differences in the RLR pathway are well-studied in ducks, there are currently few studies on TLR and NLR and their adaptor molecules in ducks during IAV infection. As these pathways converge and co-regulate each other, this is a very important piece of the story that is missing. Likewise, many proteins mentioned in this paper have been studied at the regulation level, but very few have been functionally and biochemically characterized.

We acknowledge that much of the work is yet to be done characterizing adaptor proteins in IFN and pro-inflammatory cytokine signaling networks. Investigation of these regulatory proteins in ducks and other birds, will allow us to see the conserved mechanisms, and find those that are not. Further, we acknowledge the bias that most immunological research looks at positive regulators of innate signaling. However, as ducks are equally adept at initiating and shutting down inflammatory responses, we should also begin to investigate inhibitory proteins and their expression and function. It should also be noted that functional studies in innate immunity in both ducks and chickens are limited. As such, data on PRR tissue expression and upregulation is often limited to small sample groups. Tissue expression can vary with age and breed of animals, and all studies discussed here used domestic breeds of ducks. When looking at tissue expression of genes as a potential route of resistance, it may be beneficial to also look at gene expression in wild mallards, which are constantly adapting and evolving with IAV. Indeed, it would be worthwhile understanding the allelic diversity of PRR genes and variation in function across many species of wild ducks. This may give us more insight into detection and resistance to IAV in its natural host and reservoir, the mallard duck.

## Author Contributions

LC originally drafted and edited the manuscript. KM edited the manuscript.

## Conflict of Interest

The authors declare that the research was conducted in the absence of any commercial or financial relationships that could be construed as a potential conflict of interest.
